# Glucocerebrosidase L444P mutation confers genetic risk for Parkinson’s disease in central China

**DOI:** 10.1186/1744-9081-8-57

**Published:** 2012-12-10

**Authors:** Youpei Wang, Ling Liu, Jing Xiong, Xiaowei Zhang, Zhenzhen Chen, Lan Yu, Chunnuan Chen, Jinsha Huang, Zhentao Zhang, Asrah A Mohmed, Zhicheng Lin, Nian Xiong, Tao Wang

**Affiliations:** 1 Department of Neurology, Union Hospital, Tongji Medical College, Huazhong University of Science and Technology, Hubei, 430022, China; 2 Department of Neurology, Qingdao Chengyang People’s Hospital, Shandong, 266109, China; 3 Department of Neurology, Renmin Hospital of Wuhan University, Wuhan, 430060, China; 4 Department of Psychiatry, Harvard Medical School, Harvard, USA; 5 Division of Alcohol and Drug Abuse, and Mailman Neuroscience Research Center, McLean Hospital, Belmont, MA, 02478, USA; 6 Harvard NeuroDiscovery Center, Boston, MA, 02114, USA

**Keywords:** Parkinson’s disease, Glucocerebrosidase, L444P, N370S, R120W, Central China

## Abstract

**Background:**

Mutations of the glucocerebrosidase (GBA) gene have reportedly been associated with Parkinson disease (PD) in various ethnic populations such as Singaporean, Japanese, Formosan, Canadian, American, Portuguese, Greek, Brazilian, British, Italian, Ashkenazi Jewish, southern and southwestern Chinese. The purpose of this study is to determine in central China whether or not the reported GBA mutations remain associated with PD.

**Methods:**

In this project, we conducted a controlled study in a cohort of 208 central Chinese PD patients and 298 controls for three known GBA mutations (L444P, N370S and R120W).

**Results:**

Our data reveals a significantly higher frequency of L444P mutation in GBA gene of PD cases (3.4%) compared with the controls (0.3%) (*P* = 0.007, OR = 10.34, 95% CI = 1.26 - 84.71). Specifically, the frequency of L444P mutation was higher in the late onset PD (LOPD) cases compared with that in control subjects. The N370S and R120W mutations were detected in neither the PD group nor the control subjects.

**Conclusions:**

Our observations demonstrated that the GBA L444P mutation confers genetic risk for PD, especially LOPD, among the population in the central China area.

## Background

Parkinson disease (PD) is the second most common neurodegenerative disorder. The disease is pathologically characterized by the degeneration of dopaminergic neurons in the nigro-striatal system, leading to progressive movement impairments, such as rigidity, bradykinesia, impaired balance and tremor at rest. However, the etiology and pathogenesis of this disorder are not yet thoroughly understood. Besides aging and environmental risk factors, genetic variation is also considered to be a relevant factor for PD risk. Additionally, more than 9 genes have been confirmed as PD-linked genes [1].

The Glucocerebrosidase (GBA) gene, located at chromosome 1q21, encodes the enzyme glucocerebrosidase. Mutations in the GBA gene are known to cause Gaucher disease (GD), which is an autosomal recessive glycolipid storage disorder with multisystemic manifestations [2]. It has been previously reported that parkinsonian symptoms could be observed in Gaucher patients, and increased PD incidence could be found in Gaucher families [3]. Simultaneously, increased occurrence of GBA mutations can be detected from PD brain samples [4]. Subsequently, there has been increasing documentation of the association between the GBA gene and PD in different ethnic groups, yielding contradictory results. Among these studies, the GBA mutations showed no significant difference between PD and controls in Venezuela and Norway [5,6]. However, previous studies indicate that a high ratio of GBA mutation frequency is related to increased PD risk in Singaporean, Japanese, Formosan, Canadian, American, Portuguese, Greek, Brazilian, British, Italian, Ashkenazi Jewish, southern and southwestern Chinese populations [5-20]. Specifically, three GBA mutations, L444P, N370S and R120W, accounted for about 67% of GBA mutations in Non-Ashkenazi Jewish Patients [21].

N370S is reportedly the most common mutation among Ashkenazi Jewish Patients, while it is the second most frequent mutation after L444P in Non-Ashkenazi Jewish Patients [21]. In the Asian studies, only one has demonstrated that N370S allele might be associated with LOPD in the Chinese Han population [11,13,14,16]. No R120W mutation has been detected in Ashkenazi Jewish Patient population. However, a significant association between R120W mutation and PD has been observed in Non-Ashkenazi Jewish Patients as well as in Japanese patients [21].

Overall, little is known about the correlation between these GBA gene mutations (L444P, N370S and R120W) and PD in the central China area, which includes six mostly agricultural provinces of Hunan, Hubei, Jiangxi, Henan, Shanxi and Anhui. We argue that the central China area should be of particular interest since recent epidemiological studies suggest an association of environmental toxins such as rotenone and other pesticides with the higher incidence of sporadic PD in rural areas [22-24]. As such, our study aims at examining the possible association between GBA L444P, N370S or R120W mutation and PD in 208 central Chinese PD patients and 298 controls.

## Methods

### Subjects

This study was approved by the Ethical Committee of Tongji Medical College, Huazhong University of Science (TJMC & HUST). The technology ethics and written informed consents were obtained from all subjects. A total of 208 PD patients [136 males, 72 females, mean age 56.75 ± 12.94 years, range 25 - 83] were recruited from the Department of Neurology, Union Hospital, TJMC & HUST. Diagnosis was conducted in accordance with UK-Parkinson’s Disease Society Brain Bank criteria.

We divided these patients into two groups: early onset PD (EOPD, age at onset < 50 years, 72 subjects) and LOPD (age at onset >= 50 years, 136 subjects) [25]. Concurrently, 298 controls [194 males, 104 females, mean age 51.62 ± 11.33 years, range 23 - 90] were recruited from the Department of Neurology, Union Hospital, TJMC & HUST (see Additional file 1 for the demographic information of patients and controls). The controls were selected from subjects whose physical examination revealed no evidence of PD, without a family history of PD, and no evidence of Alzheimer’s disease, cerebrovascular disease or any other neurological disorder. There is no blood relationship between controls and patients. All PD and control individuals are residents of the central China area.

### Genetic analysis

Mutation screening was performed using the polymerase chain reaction-restriction fragment length polymorphism (PCR-RFLP) method. Peripheral blood (5 ml) was collected from each participant and placed in EDTA-containing vacuum tubes. Genomic DNA was extracted using the phenol chloroform extraction method, stored at -20°C to await further processing. The optional parameters for 25 μl PCR reaction system were 12.5 μl 2 x Power Taq PCR MasterMix (Bioteke Corporation, Beijing, China), 1 μl primers (10 pmol/μl), 1 μl DNA templates (30 ng/μl) and 9.5 μl ddH2O. The L444P, N370S and R120W primers were obtained from previous studies [15,17]. The PCR products were digested overnight with NciI, XhoI, NciI, respectively, at 37°C, with the effective digestion system of 15 μ1 including 10 μl DNA solution and 10 U enzyme.

The digestion product was electrophoresed on 2% agarose gel and visualized by Sybr-greenI. The L444P and N370S mutations create split clips with the use of the NciI and XhoI restriction enzymes, respectively. The R120W mutation cancels a native restriction site for Ncil. All mutated alleles were notarized by DNA direct sequencing of PCR products from a second amplification. The DNA sequences were analyzed using Chromas, Version 2.22.

### Statistical analysis

Statistical differences of carrier rates among PD and control groups were analyzed by means of the Fisher test. The odds ratio of different alleles across EOPD and LOPD groups were analyzed with both the Mantel-Haenszel test and meta-analysis. The difference of age at onset between carriers and non-carriers or total patients was analyzed by using the two tailed Student’s test. Odds ratio (OR) with 95% confidence intervals (95% CI) was employed to test the association between the GBA mutation and PD. Results were presented as mean ± standard error. P < 0.05 is considered to be statistically significant.

## Results

### The GBA L444P mutation is correlated with *an* increased PD risk

In 208 PD patients, GBA mutations were found in 7 (3.4%) patients by PCR-RFLP (Additional file 2) and confirmed by DNA sequencing (Table 1 and Additional file 2B and 2C). The results of the PCR-RFLP analysis and DNA sequencing were in agreement (Additional file 2). Moreover, all GBA mutations were heterozygous for L444P. Among the 298 control subjects, only 1 (0.3%) had a mutation, and this mutation was also heterozygous for L444P. No N370S or R120W mutation was detected in any PD patient or control subject. The difference of GBA L444P mutation frequencies in PD patients and control subjects was statistically significant (* *P* = 0.01). Moreover, there was a correlation between increased PD risk and higher GBA L444P mutation (OR = 10.34, 95% CI: 1.26 - 84.71). The distribution*s* and association of GBA mutations in patients and controls are shown in Table 1.

**Table 1:** Distributions and association of GBA mutations in patients and controls

**Mutation**	**PD(n/N)**	**Controls(n/N)**	**Odds ratio**	**95% CI**	**p value**
L444P	7/208(3.4%)	1/298(0.3%)	10.34	1.26-84.71	0.01*
N370S	0/208(0)	0/298(0)			
R120W	0/208(0)				

*Fisher’s exact test.

### Higher frequency of L444P mutation is associated with LOPD

Following age-stratification, a significantly higher frequency of L444P mutation was found in LOPD cases compared with that in controls (OR = 13.71, 95% CI: 1.63 - 115.00, *P = 0.005, Table 2). However, no detectable difference was observed between EOPD subjects and controls (OR = 4.18, 95% CI: 0.26 - 67.69, *P* = 0.352, Table 2).

**Table 2:** Differences in age-stratification between mutation carriers and non-carriers

**Subgroup**	**PD (n/N)**	**Controls (n/N)**	**Odds ratio**	**95% CI**	**p value**
EOPD	1/72	1/298	4.18	0.26-67.69	0.352
LOPD	6/136	1/298	13.71	1.63-115.00	0.005*

*Fisher’s exact test.

### Gender or age distribution of mutation carriers and non-carriers showed no difference

Among all PD patients, no significant difference in age was found between the L444P mutation carriers and non-carriers. The average age was 56.14 ± 8.34 years in the carriers and 56.78 ± 13.08 in the non-carriers (*P* = 0.899, Table 3). Following age-stratification, no difference was observed between carriers and non-carriers in LOPD cases (58.00 ±7.38 years vs. 64.73 ± 8.21 years; *P* = 0.051, Table 3) and EOPD subjects (45.00 years vs. 42.21 ± 5.66 years, *P* = 0.626, Table 3). There was no significant difference observed in gender between mutation carriers and non-carriers (*P* = 0.732, Table 3).

**Table 3:** Age at onset and gender of PD patients

	**Carriers**	**Non-carriers**	**p value**
Age at onset (year)			
PD	56.14±8.34	56.78±13.08	0.899
LOPD	58.00±7.38	64.73 ± 8.21	0.051
EOPD	45.00	42.21 ± 5.66	0.626
Gender			
Male (%)	5(71.4)	131(65.2)	0.732
Female (%)	2(28.5)	70(34.8)	

## Discussion

Central China is relatively characterized by the six provinces of Hunan, Hubei, Jiangxi, Henan, Shanxi and Anhui, all largely agricultural. Epidemiological study suggests that living in a rural area, drinking well water, farming and exposure to pesticides may be significant risk factors in the development of PD [21]. Central China is considered to be the cradle of agriculture in China. Importantly, the population density in this region is very high. Previous epidemiological study also showed that the prevalence of PD in South-Central (including Central China area) China is higher compared to other areas of China [21]. In this study, we investigated the association of 3 GBA gene mutations (L444P, N370S and R120W) with central Chinese PD patients. The frequency of the GBA mutations in PD patients was 3.4%. This statistically significant difference in GBA mutations between PD patients and control subjects suggests that the GBA gene is a susceptibility gene in central China and GBA mutation may increase the risk of PD. High frequency of GBA mutations was found in LOPD cases. There was no significant difference due to gender between mutation carriers and non-carriers. Additionally, the average age at onset of PD carriers between carriers and non-carriers was insignificant.

GBA L444P mutation has reportedly been associated with PD in various ethnic groups [7,9-16,19,20,26]. In a previous study, L444P was identified as the most frequent mutation in non-Ashkenazi Jewish patients [21]. However, there was no significant difference in PD patients and controls in other ethnic groups diagnosed with the L444P mutation (Toft and others 2006; Eblan and others 2006). In this study, the GBA mutations of PD subjects were all heterozygous for L444P, and there was a significant difference between the results of PD patients and controls. Together with the results of two other studies in Southern and Southwestern regions of China [10,13], these data provide evidence that the L444P mutation confers risk for PD in China. Furthermore, following age-stratification, this study revealed a higher frequency of the GBA L444P mutations in LOPD patients compared with that in the control group. This result is different from that of other studies which showing GBA gene mutations occurred in EOPD rather than LOPD [10,21]. The discrepancy may be attributed to different genetic backgrounds or environmental factors among others.

The N370S mutation, a common mutation in Jewish and non-Jewish GD patients, is rarely presented in Asian patients [27]. Similar to previous findings, our result showed that N370S mutation was absent in both PD patients and control subjects [13,21]. A recent study indicated that N370S might affect age of onset and may also contribute to LOPD susceptibility in Chinese Han populations [8]. There were several explanations for the various research results, including the large influx of a Jewish population to China during World War II, and the possible introduction of the gene mutation by traders using the Silk Road in the distant past [27]. It is also conceivable that the N370S mutation may exist only in specific areas of China and/or some other Asian regions. More research will be necessary to address the question of whether the mutations exist in different areas of China.

R120W is considered as the third most common mutation of non-neuropathic GD. A previous Japanese study determined that R120W was prevalent and significantly associated with PD (15/534, *P* < 0.001) [11]. A Taiwan study found one R120W mutation in PD patients as well [15]. However, the association between PD and the GBA R120W mutation was not significant. No R120W mutation has been found in the Ashkenazi Jewish PD patient population. Our study was the first to explore the association between the R120W mutation and PD in Mainland China. Similar to the case of the Ashkenazi Jews, no R120W mutation was found in our PD patients or controls [11,15]. Different ethnic groups and environmental factors among different areas may explain the discrepancy.

The L444P mutation, resulting from a T to C transition in nucleotide 6092 of the GBA gene, can make leucine into proline, which leads to the imperfection of glucocerebrosidase. The mechanism of GBA mutations conferring risk for PD is not yet completely understood. First, decrease in glucocerebrosidase activities has a role in the pathogenesis of PD [11]. Second, GBA mutations contribute to protein misfolding [28], leading to lysosomes and ubiquitin-proteasome system dysfunction which cause neurodegeneration [29]. Third, GBA mutations may potentially accumulate the substrates glucocerebroside and/or glucosylsphingosine, disrupting membrane binding of alpha-synuclein, which prevents the formation of fibrillar protein structures of alpha-synuclein [29,30]. Finally, the fluctuation in the levels of ceramide, the result of homozygous or heterozygous GBA mutations, is considered to cause Parkinsonism as well [18]. More research evidence is needed to confirm the mechanism of these hypotheses. Potential molecular therapies and genetic counseling of patients and family members may be developed for PD patients with GBA mutations once the mechanism for the association is determined.

Additionally, we acknowledge that our case–control result may be susceptible to bias because of the test method and sample size. In our study, we chose to screen mainly by restriction digest rather than by sequencing. It has been reported that as many as 45% of mutant alleles can be missed among non-Ashkenazi Jewish patients (20% among Ashkenazi Jewish patients) when screening for only the N370S and L444P mutations instead of the whole gene [21]. Replication of genetic associations in various populations is essential to establish specific genetic contribution to the disease. The purpose of this study is to confirm the contribution of GBA mutations to PD etiology, although we may miss some mutant alleles by restriction digest for only three mutations. Moreover, given the small sample size (208 cases and 298 controls, 136 LOPD and 72 EOPD), replications of the positive associations in the Chinese population is warranted. We will increase the sample size and explore new GBA gene mutations by sequencing the whole gene in the next step.

## Conclusions

In summary, we presented GBA L444P mutation as a relatively prevalent genetic risk factor for sporadic PD. GBA may act as a novel therapeutic target for PD and may be used in the early diagnosis of PD in the future. With further research, we expect to be able to provide new avenues for developing therapeutic alternatives for PD based on the GBA gene.

## Abbreviations

GBA: Glucocerebrosidase; PD: Parkinson’s disease; LOPD: Late Onset of Parkinson’s Disease; EOPD: Early Onset of Parkinson’s Disease; GD: Gaucher disease; TJMC & HUST: Tongji Medical College, Huazhong University of Science; PCR-RFLP: Polymerase chain reaction-restriction fragment length polymorphism; OR: Odds ratios.

## Competing interests

The authors declare that they have no competing interests.

## Authors’ contributions

YW, LY, JX, XZ, ZC, LY, CC, JH, ZTZ, AM, ZCL, NX, TW contributed to the conception and design. YW, LY, JX, NX, XZ, ZC, LY, CC took care of the PCR studies. YW, LY, JX, LY, CC, JH, ZTZ, NX, TW analyzed and interoperated the data. YW, LY, JX, ZCL, NX, TW, AM coordinated all the experiments and helped to draft the manuscript. All authors read, revised and approved the final manuscript.

## Supplementary Material

Additional file 1 The demographic information of patients and healthy controls. Click here for file

Additional file 2 **PCR analysis and Sequence of the L444P mutation in Patients with PD.** (A) The restriction enzyme NciI digests a 638 bp PCR product, producing two fragments of 536 bp and 102 bp. The digestion of wild-type PCR product and L444P mutation with a Low Range DNA Ladder are shown in (B) and (C). (B) Normal Sequence of the GBA gene; (C) The L444P Mutation in the GBA gene. The arrows indicate the position of the mutation. Click here for file
