# Supplementary figures and images for: Glucocerebrosidase L444P mutation confers genetic risk for Parkinson’s disease in central China
Source: Behav Brain Funct. 2012 Dec 10;8:57. doi: 10.1186/1744-9081-8-57 (PMC3538614; doi:10.1186/1744-9081-8-57)

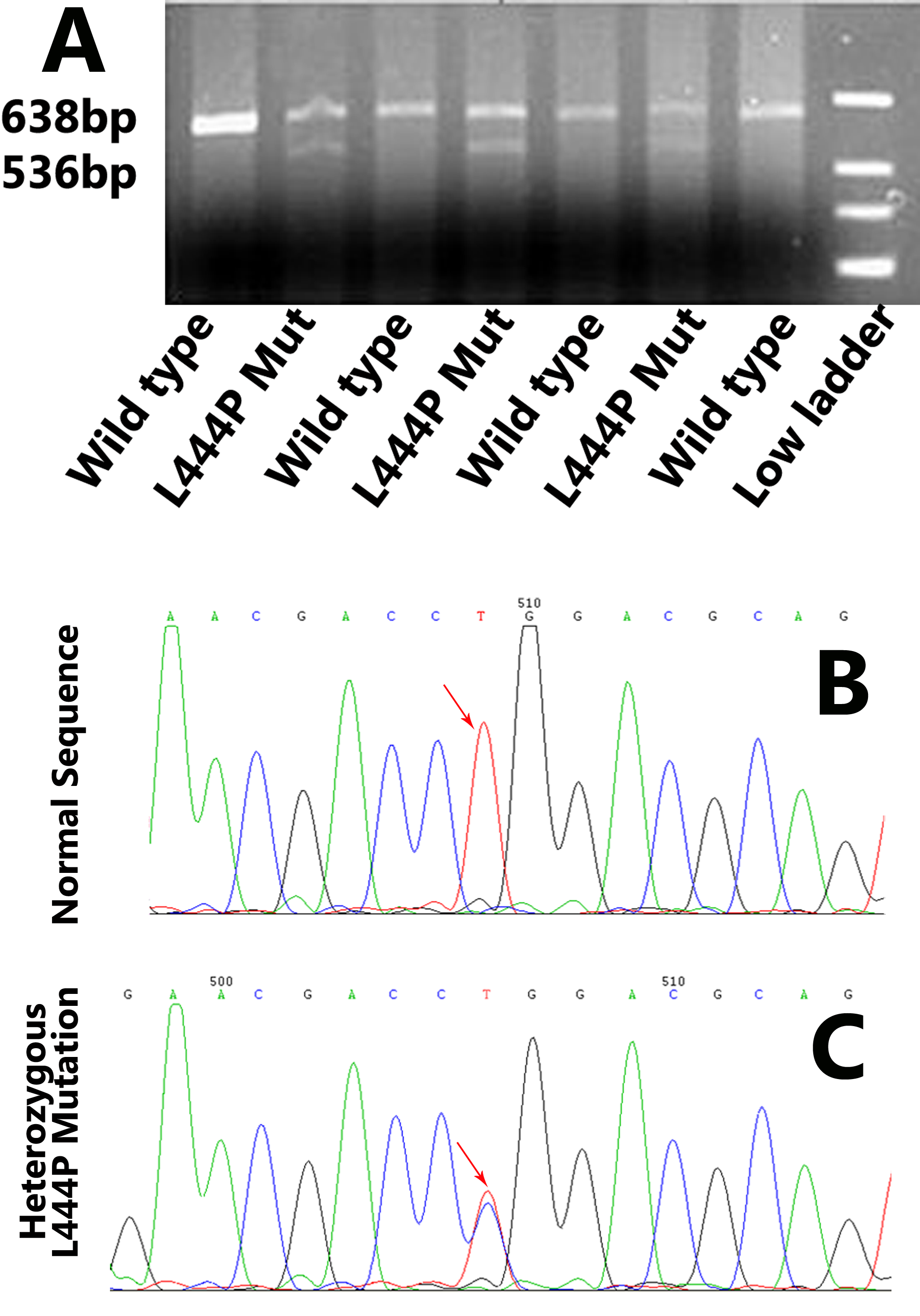

Supplement: Additional file 2 — PCR analysis and Sequence of the L444P mutation in Patients with PD. (A) The restriction enzyme NciI digests a 638 bp PCR product, producing two fragments of 536 bp and 102 bp. The digestion of wild-type PCR product and L444P mutation with a Low Range DNA Ladder are shown in (B) and (C). (B) Normal Sequence of the GBA gene; (C) The L444P Mutation in the GBA gene. The arrows indicate the position of the mutation. [file 1744-9081-8-57-S2.tiff]
